# Skin Lesions in Swine with Decompression Sickness: Clinical Appearance and Pathogenesis

**DOI:** 10.3389/fphys.2017.00540

**Published:** 2017-07-25

**Authors:** Long Qing, Dinesh K. Ariyadewa, Hongjie Yi, Yewei Wang, Quan Zhou, Weigang Xu

**Affiliations:** ^1^Department of Diving and Hyperbaric Medicine, Faculty of Naval Medicine, Second Military Medical University Shanghai, China; ^2^Department of Medicine, Sri Lanka Naval Headquarters Colombo, Sri Lanka

**Keywords:** decompression illness, Bama swine, cutis marmorata, autochthonous bubbles, veneous bubbles, arterial bubbles

## Abstract

Skin lesions are visual clinical manifestations of decompression sickness (DCS). Comprehensive knowledge of skin lesions would give simple but strong clinical evidence to help diagnose DCS. The aim of this study was to systematically depict skin lesions and explore their pathophysiological basis in a swine DCS model. Thirteen Bama swine underwent simulated diving in a hyperbaric animal chamber with the profile of 40 msw-35 min exposure, followed by decompression in 11 min. After decompression, chronological changes in the appearance of skin lesions, skin ultrasound, temperature, tissue nitric oxide (NO) levels, and histopathology were studied. Meanwhile bubbles and central nervous system (CNS) function were monitored. All animals developed skin lesions and two died abruptly possibly due to cardiopulmonary failure. A staging approach was developed to divide the appearance into six consecutive stages, which could help diagnosing the progress of skin lesions. Bubbles were only seen in right but not left heart chambers. There were strong correlations between bubble load, lesion area, latency to lesion appearance and existence of cutaneous lesions (*P* = 0.007, *P* = 0.002, *P* = 0.004, respectively). Even though local skin temperature did not change significantly, skin thickness increased, NO elevated and histological changes were observed. Increased vessel echo-reflectors in lesion areas were detected ultrasonically. No CNS dysfunction was detected by treadmill walking and evoked potential. The present results suggest skin lesions mainly result from local bubbles and not CNS injuries or arterial bubbles.

## Introduction

Since decompression sickness (DCS) was first identified among caisson workers, “skin bends” have been recognized as a symptom of mild DCS (DCS Type I) (Mitchell et al., 2004). Diagnosis of DCS depends upon clinical manifestations including skin lesions. Even with skin lesions being simple and, in some instances, the first sign to appear at the onset of DCS in divers, limited studies have been conducted to describe their temporal development. Itching or painful non-elevated red-bluish spots, erythema that progresses to mottling or marbling and livedo-reticularis are descriptions of skin lesions that appear with DCS (Dennison, 1971; Buttolph et al., 1998; Gibbs et al., 2005). Descriptions in different studies, however, in general do not systematically depict the evolution of the appearance of lesions, which could help identify the underlying pathogenesis.

The pathogenesis of skin lesions still has not been clarified. It is widely accepted that rapid decompression generates sub-cutaneous bubbles which leads to a cascade of reactions and sometimes ultimately produces cutis marmorata (CM) (Hugon, 2014). But no direct evidence has proven whether local inert gas bubbles cause DCS skin lesions. One recent hypothesis suggested that CM skin DCS was associated with brainstem bubble embolization which damaged the autonomic nervous system regulating skin blood vessel dilation and constriction (Germonpre et al., 2015). Another study failed to find the relationship between CNS deficits and skin lesions (Broome and Dick, 1996). One more hypothesis is that arterial bubbles embolize in the skin as the result of a right-to-left shunt in the heart (Wilmshurst et al., 2001). Whether the pathogenesis of skin lesion DCS involves autochthonous or arterial bubbles, or CNS injury, remains unclear.

Comprehensive knowledge of skin lesions appearing with DCS could assist diagnosing DCS. Based on the swine DCS model of a prior experiment, the purpose of this study was to explore the time course of the appearance of skin lesions and the basic causes, toward increased understanding of the underlying pathogenesis.

## Materials and methods

### Animals

Ethical clearance for this study was obtained from the Ethics Committee for Animal Experiment of the Second Military Medical University. The experiment protocol was carried out in accordance with internationally accepted humane standards (Russell and Burch, 1959).

Thirteen Bama swine were received by the animal husbandry facility of the university. They were males, castrated 2 weeks after they were born to avoid the influence of hormonal effects. The animals were allowed to acclimatize individually to general laboratory temperature of 23 ± 1°C and humidity of 50–65% and provided standard laboratory swine meal, in portions of 2% of their body weight daily and water was made available *ad libitum*. They weighed between 20 and 25 (22.0 ± 1.2) kg at the beginning of the experiment and were confirmed free of systemic infections or skin diseases.

### Experimental procedure

The animals were singly utilized in the experiment. Four days before hyperbaric exposure, they were trained to walk on a treadmill once a day for 2 days. Two days before hyperbaric exposure, evoked potentials were measured and normal skin samples were collected. One day prior to hyperbaric exposure, a thorough skin examination was performed to find contusions, abrasions and lacerations and the hair of all chosen sites was trimmed. Structure and blood flow of heart were measured. One hour before the exposure, skin temperature, and cutaneous ultrasound (for structure and blood flow) were recorded at trimmed sites.

### Hyperbaric exposure

The swine were placed one at a time in a 1,000 L animal compression chamber (DCW150, Yangyuan, Shanghai, China) with view ports to observe the animal inside. The chamber was pressurized with air to 40 msw and maintained for 35 min before decompression. Compression was performed in 5 min which began at 5 msw/min to minimize the possible discomfort to the swine. Chamber oxygen (O_2_), carbon dioxide (CO_2_), temperature, and relative humidity were continuously monitored and maintained at 21%, <0.3%, 22–24°C, and 65–75%, respectively. Decompression was conducted in linear segments of 5 msw/min from 40 to 30 msw, 4 msw/min from 30 to 20 msw, 3.3 msw/min from 20 to 10 msw, and 2.9 msw/min from 10 to 0 msw.

### Skin lesion observation

**Figure 1:**
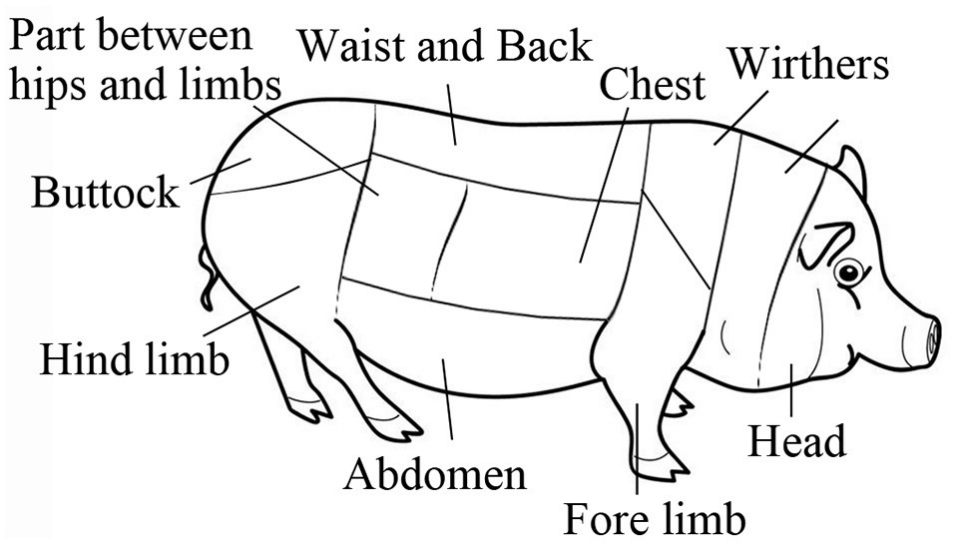
Body skin map of a swine.

After surfacing, the skin was thoroughly examined for up to 36 h. The observations were continuous in the first 6 h and periodic with 1 h in next 18 h. In the last 12, 2–3 h interval of observations was arranged. The appearance, evolution, locations, and dimensions of lesions were recorded on a swine-shape figure (Figure 1). Lesions were measured by the palm of a single experimenter, which is similar to the estimation of burn surface area. The swine body surface area was calculated by Meeh-Rubner equation Area = 0.0974 × Weight2/3 (Quiring, 1955).

### Cutaneous temperature and ultrasound detections

Based on our previous experimental results, several sites were selected as main observational regions including the four limbs, neck, chest, abdomen, waist, and back. Skin temperature was recorded using infrared thermometer (JXB182, Berrcom, China) with 0.1°C resolution. Skin thickness from the keratin cell layer to the dermis layer was measured using an Apogee 1000 ultrasound unit and a 14 MHz transducer (L8L38C, SIUI, Shantou, China) in color Doppler mode to image skin vessels. Both measurements were repeated during the evolution of lesions.

### Heart structure and blood flow

Heart structure was evaluated using transthoracic echocardiography (TTE) (P3f14C, SIUI, Shantou, China). A left ventricular long axis view was obtained, and the probe position was then adjusted to obtain an apical four chamber view. In the latter view an examination (incorporating observation of gross structure and use of color flow Doppler) was made for structural abnormalities in the atrial and ventricular septum. Although no formal bubble contrast studies were conducted, the real time repeat observations of venous and arterial bubbles after decompression served as an additional check for the presence of right to left shunt.

### Bubble detection

Bubbles in heart chambers were detected extrathoracically by the same machine and detector described above. Detection was repeated at 30, 60, 90 min, 2, 3, 4, and 6 h following surfacing, each lasting for 2 min. Left ventricular long axis view was found as the datum plane first, in which left atrium (LA), left ventricle (LV), and aorta (AO) can be seen clearly. Then the probe was adjusted to the aortic root short axis as the final view for detection. In this view, right ventricular outflow tract (RVOT), pulmonary artery (PA), and AO were presented. No detection was performed after 6 h due to the necessary oxygen breathing during anesthesia for evoked potential detection. Bubbles in ultrasound images were scored by Eftedal-Brubakk grading scale (Eftedal and Brubakk, 1997).

### Treadmill training and motor ability test

The two training sessions before hyperbaric exposure were defined as complete when each animal walked comfortably on a treadmill (YS-C600, KunYu, Henan, China) for 5 min at 1.6 km/h. At 2 and 6 h post-dive, motor function were tested on the same treadmill. The speed was gradually increased to 1.6 km/h at 0.4 km/h increments over 30 s, and the animal walked for 5 min at the highest speed, which earned a score of 5. If the animals could not stand or the endpoint on the treadmill was not achieved, the 5-point Tarlov score was adopted to grade motor function (Mahon et al., 2010).

### Detection of evoked potentials

Evoked potential detection was performed 2 days prior to, and 6 h after, the hyperbaric exposure. Animals fasted for 12 h before anesthesia, which was induced by intramuscular injection of 0.05 mg/kg atropine and 0.1 ml/kg Sumianxin and maintained with inhaled isoflurane (6%) via endotracheal intubation and an anesthetic machine (WATO EX-20 Vet, Mindray, Shenzhen, China). Sensory evoked potential (SEP) was measured using electromyography and evoked potential instruments (NDI-094, Haishen, Shanghai, China). Stimulating electrodes were placed into the right and left ankles to stimulate tibial nerves and electroneurographic signals were collected from first lumbar vertebra and head by receiving electrodes, which were defined as spinal somatosensory evoked potential (SSEP) and cortical somatosensory evoked potential (CSEP), respectively.

### NO and pathological examinations

After each evoked potential detection with the animal still under anesthesia, skin samples were collected from selected areas and the center of stage IV lesions (described in Results)/adjacent non-affected sites. Biopsy sites were cleaned thoroughly with povidone iodine and saline. Full thickness skin biopsies were obtained, 3 × 1 cm spindle shaped. Excess fat from the biopsy was removed and 0.5 g of skin tissue separated from each specimen was stored at −80°C prior to NO determination using a total nitric oxide assay kit (Beyotime, Shanghai, China). The rest of the skin was fixed in 10% formaldehyde solution for 48 h before being mounted in paraffin, longitudinally sectioned, stained with Haematoxylin and Eosin (HE), and observed and photographed using a microscope (Eclipse55i, Nikon, Japan).

### Statistical analysis

All data are presented as mean ± *SD*. The Pearson correlation coefficient was derived to evaluate the relation between bubble load, skin lesion area, and latent time to appearance. The changes in skin temperature and thickness were tested using one-way ANOVA followed by *post-hoc* Student Newman–Keuls test. The changes in NO level were tested by Dunnett's test. Latent time in SEP was tested for significance with a two-tailed Student's *t*-tests. Significance in all cases was accepted at *P* ≤ 0.05.

## Results

### Cutaneous appearance

**Figure 2:**
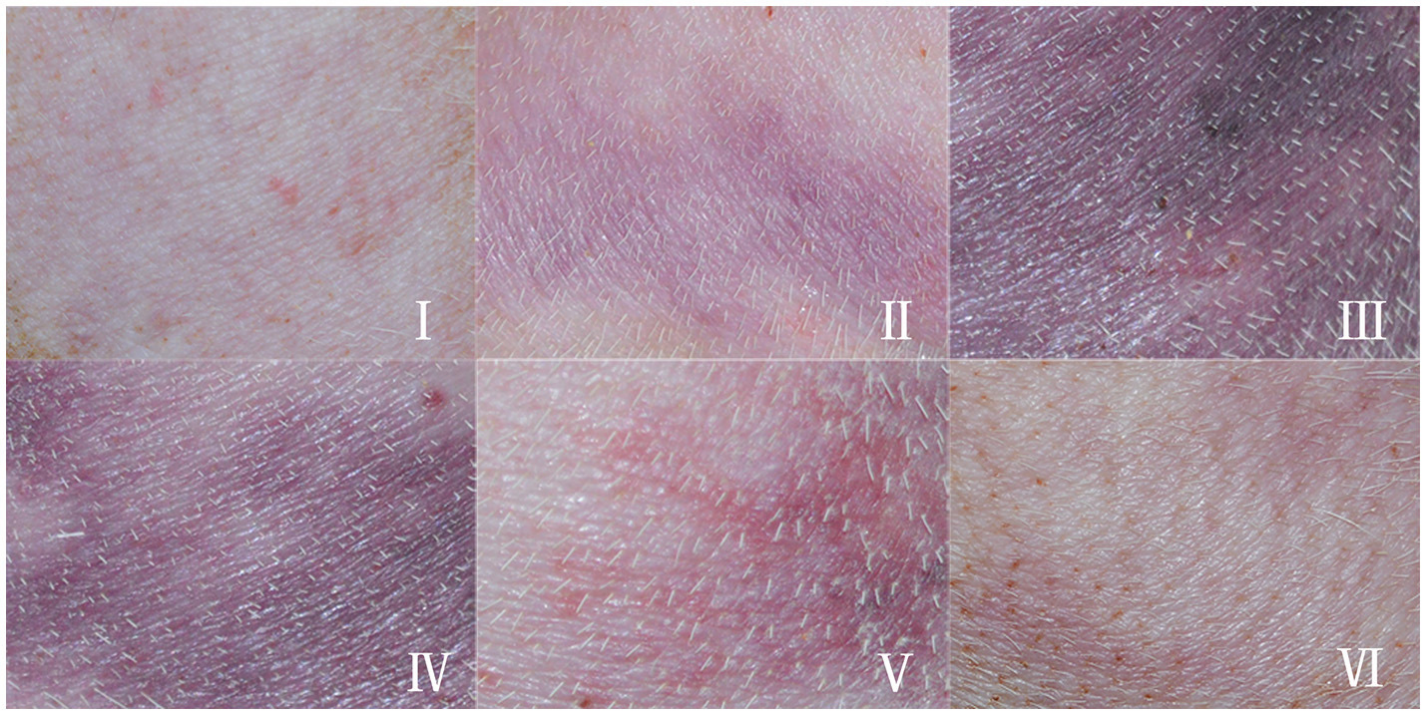
Typical appearance of skin lesions in a swine DCS model. Swine were subjected to a simulated air dive to 40 m for 35 min with a decompression in 11 min, skin appearance were observed and recorded. The hair had been removed prior to the experiment. **(I)**: Erythematous skin, erythematous skin with purple-red discrete macular lesions; **(II)**: Marbling, evolving purple-red marbling; **(III)**: Homogenous purple-red lesion, purple-red homogenous, macular lesion; **(IV)**: Fading lesion, purple-red or blue-black shrinking, and fading macular lesion with reappearance of marbling; **(V)**: Scattered lesion, blue-black scattered, and fading macular lesion; **(VI)**: Remnant, faintly visible blue-black discrete macular lesion.

Eleven swine survived the experiments, and all developed skin lesions. The lesions showed obvious temporal patterns of change. The erythema, homogeneous purple-red color of the lesion gradually progressed into patterns of marbling or scattered lesions and completely disappeared without leaving any residual macroscopic skin changes. We therefore proposed a graduated system for DCS skin lesions. Not all lesions displayed each feature in the sequence, but typical lesion development can be described using stages shown in the caption of Figure 2, and chronological, exemplary appearances are shown in Figure 2.

Each swine had at least two lesions located in different sites along the body. All stages appeared in most of the lesions. In each lesion, stage III was the most serious appearance and the area was measured. 10–20% lesions in each animal were mild, the lesions faded quickly within 1 h after Stage I and II. Hence, the mild lesions did not show Stage III manifestation and were not counted in the total lesion area. Occurrence of Stage III lesions in the body sites are listed in Table 1.

**Table 1:** Occurrence of skin lesions in the body sites in a swine DCS model.

**Body sites**	**Occurrence**
Hind limb	10/11
Waist and back	8/11
Abdomen	8/11
Chest	7/11
Fore limb	6/11
Neck	5/11

*Eleven swine following a simulated air dive to 40 m for 35 min with 11 min decompression were observed for skin lesions over the body. Only Stage III lesions (see in text) were counted*.

**Figure 3:**
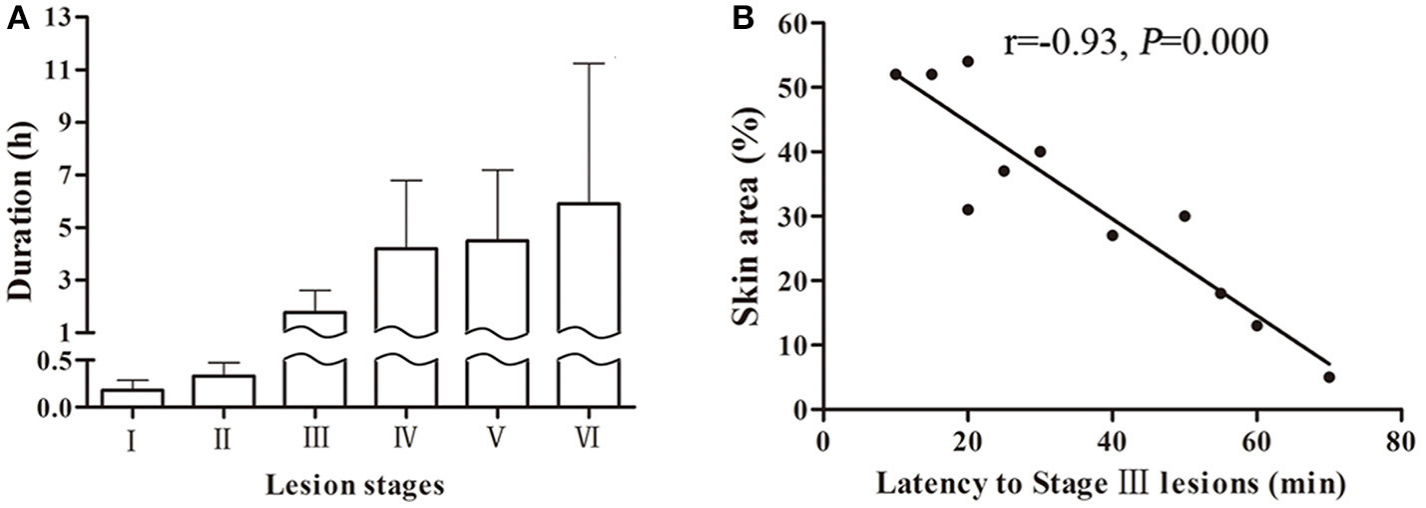
Skin lesion durations of each stage in a swine DCS model. Eleven swine following a simulated air dive to 40 m for 35 min with 11 min decompression. A total 44 lesions were observed. Durations of each stage are shown **(A)**. The correlation between skin area and latency to Stage III lesions are shown **(B)**.

Skin lesions appeared quickly following surfacing (13 ± 12 min) and almost simultaneously in different body sites. The speed of developing into stages varied among animals and lesions. Stage I and II usually existed for a relatively short time only, while stages III–VI took longer to evolve. Some lesions, especially in dorsal skin, remained at stage IV even after 24 h from surfacing. The mean duration for each stage is shown in Figure 3A from the 44 lesion s listed in Table 1, which show considerable variation. Another five lesions were distributed in sites other than the selected and trimmed areas.

The latency to stage III lesion can be taken as a parameter to reflect lesion severity as it correlates well with lesion area. As shown in Figure 3B, the shorter the latent time to stage III lesion, the larger the lesion area (*r* = −0.93, *P* = 0.000).

### Heart structure and blood flow

From the apical four chamber view, all swine had complete structure of ventricular septal and interatrial septum, and no patent foramen ovale and no abnormal blood flow were observed.

### Bubble scores and correlation with skin lesions

**Figure 4:**
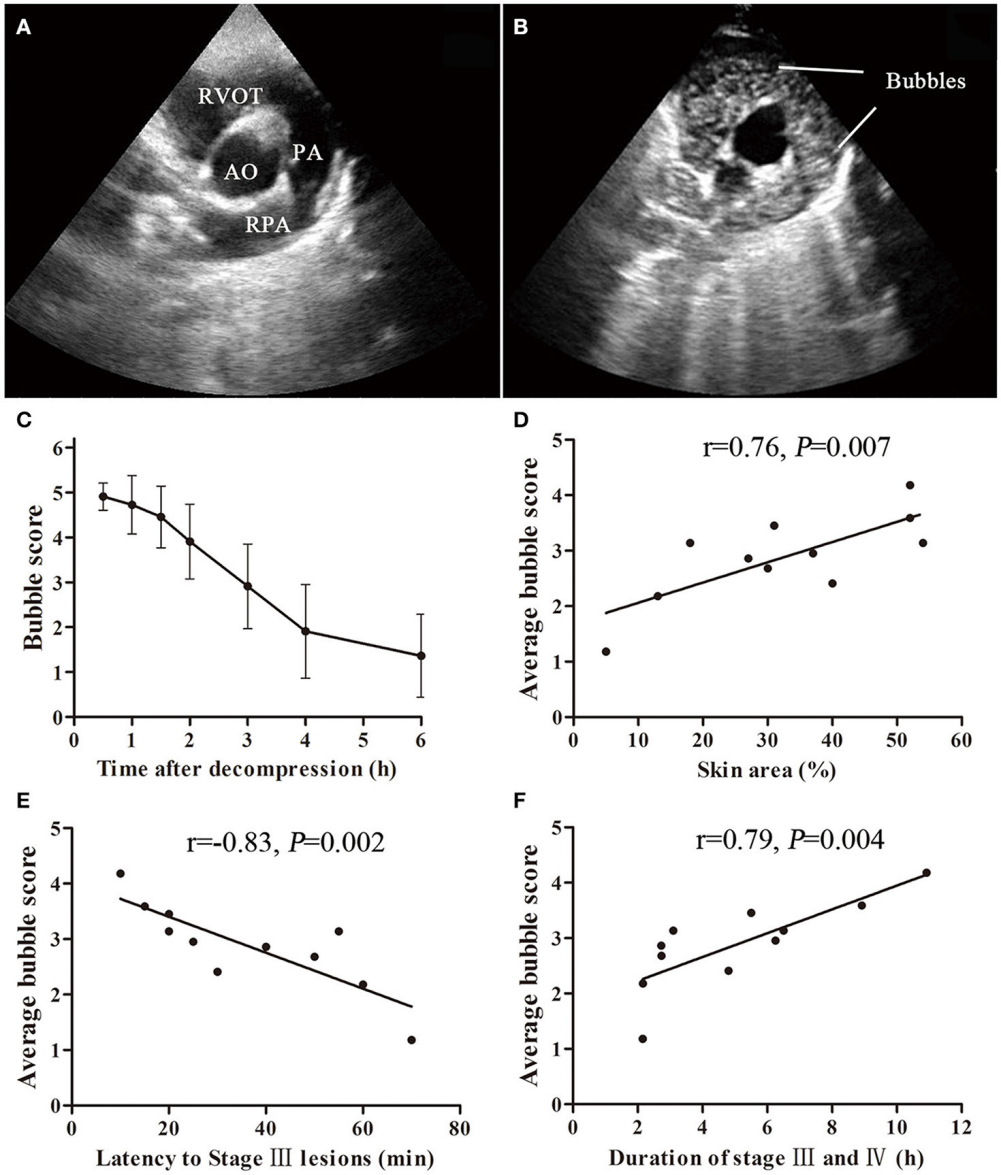
Bubble formation and correlation with cutaneous lesions in a swine DCS model. Ultrasound bubble detection was performed on 11 swine after a simulated dive to 40 m-35 min with a rapid decompression. An aortic root short axis view was chosen, which shows aorta (AO), right ventricular outflow tract (RVOT), pulmonary artery (PA) and right PA (RPA) **(A)**. Bubbles can be seen in RVOT and PA **(B)**. The bubbles were scored by EB grading scale. Bubble amount decreased slowly during the detecting period from 0.5 to 6 h after decompression **(C)**. Significant correlations between average bubble loads and latency to Stage III lesions, skin area (lesion area/whole body area) or duration of stage III and IV are shown **(D–F)**.

Figure 4 shows bubbles and the scores detected from the right hearts in the 11 survivors. Gas bubbles could be clearly observed (moving bright spots) in right ventricular outflow tract RVOT and PA in ultrasound images. No bubbles were observed in AO, LV, and LA in all the surviving swine. During the observation period, bubble amount was greatest at the first detection performed at 30 min following decompression, which showed a white-out image that single bubbles could not be discriminated in most of the swine. Bubble grades gradually decreased and disappeared within 6 h in 9 out of the 11 swine. The average bubble score for each pig was calculated from the area under the curve (similar to that shown in Figure 4C) divided by 6 h, and was regarded as bubble load for each animal.

There were significant correlations between bubble load and maximum lesion area ratio, latency to stage III lesion or total duration of stage III and IV. As shown in Figures 4D–F, the heavier the bubble load, the higher the lesion area ratio (*r* = 0.76, *P* = 0.007), the earlier the occurrence of stage III lesions (*r* = −0.83, *P* = 0.002), and the longer existence of cutaneous lesions (*r* = 0.79, *P* = 0.004). According to the area and latency of Stage III lesions, the bubble grade could be estimated (Table 2).

**Table 2:** Grading of skin lesions according to lesion area (x ± *SD*).

**Lesion area (%)**	***n***	**Latent time to stage III lesion (min)**	**Average bubble grade**
<30%	4	56.3 ± 12.5	2.3 ± 0.9
30%~50%	4	31.3 ± 13.2	2.9 ± 0.5
>50%	3	15.0 ± 5.0	3.6 ± 0.5

*Skin lesions were observed in 11 swine decompressed from a simulated air dive to 40 m-35 min. Circulating bubbles were detected and scored. According to the area and/or latency to Stage III lesion, the bubble grade could be estimated*.

### Skin ultrasound findings

**Figure 5:**
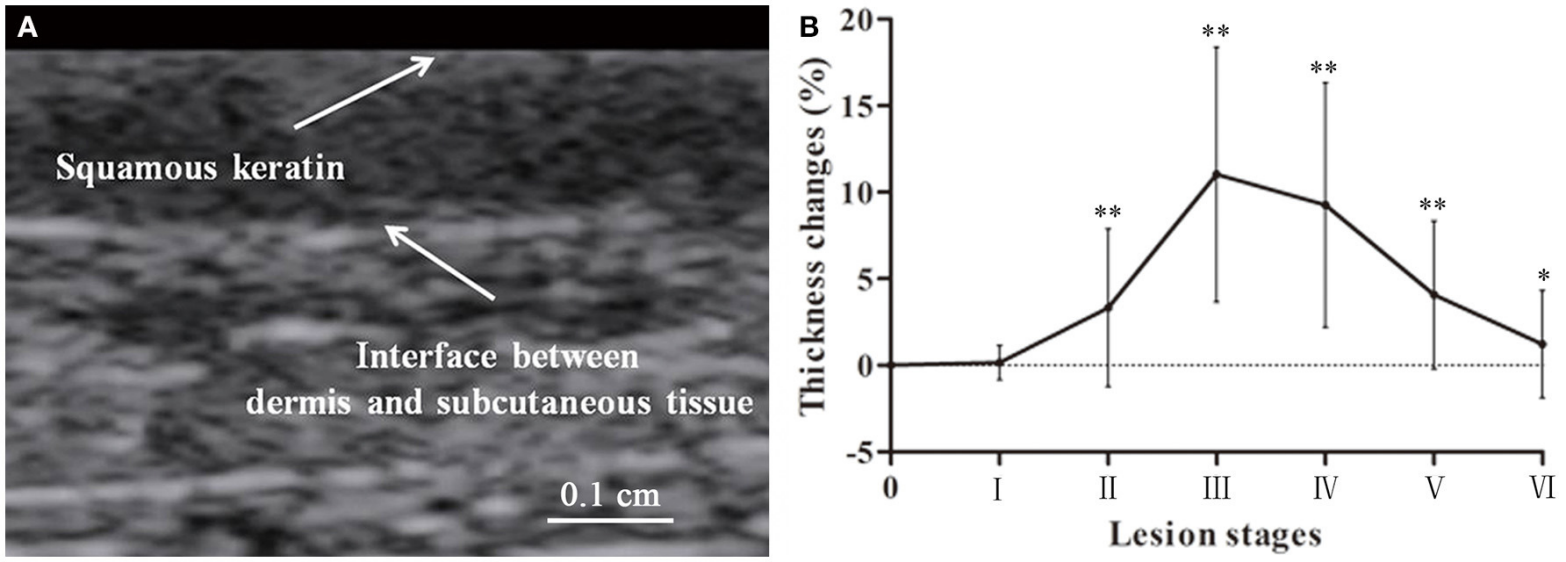
Lesion skin thickness in a swine DCS model. Thickness of skin was determined from the squamous keratin layer to the dermis **(A)**. Forty four lesions were measured. Thickness changes in percentage to the normal are presented as mean ± *SD* (Compared with normal control ^*^P < 0.05, ^**^P < 0.01) **(B)**.

Swine skin thickness from the squamous keratin layer to the dermis varied across body areas including the thinnest abdomen (1.4 ± 0.1 mm) and thickest waist and back (3.7 ± 0.2 mm). Figure 5 shows the change in lesions with stages, increasing before Stage III and a gradual recovery from Stage IV to VI. The maximum thickness appeared around stage III to IV.

**Figure 6:**
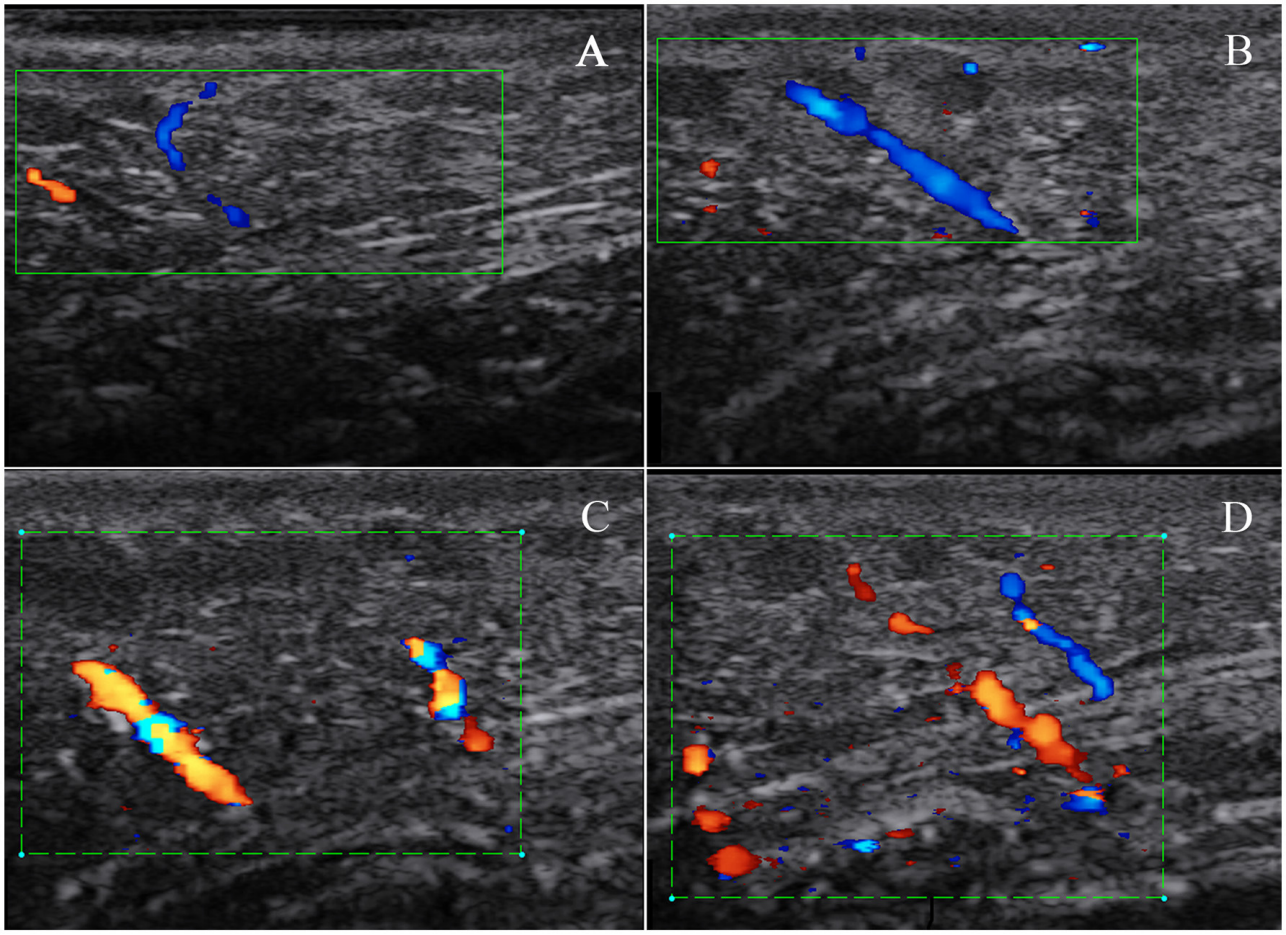
Subcutaneous blood flow in a swine DCS model. The swine was subjected to a simulated air dive to 40 m for 35 min with an 11 min decompression. Skin blood flow was detected by color Doppler scanning on normal skin in the lateral neck before the dive and repeated on a lesion in the same location. Only two small vessels in subcutaneous tissue were found in normal skin **(A)**. More apparent blood flow signals in the same site were detected on a Stage III lesion, and images in **(B–D)** show the changes in blood flow signals at different probe angles during detection.

Generally, vessels could not be detected by color Doppler scanning in skin layer and were also hardly to be found in subcutaneous tissue. In lesion skin, especially in Stage III, in the lateral neck subcutaneous tissue, vessel images were not grossly apparent (Figure 6).

### Skin temperature

Surface temperature was slightly different in different body areas. However, changes showed no statistically significant variation during the six stages on all lesion surfaces (*P* = 0.880).

### Skin tissue NO level

**Figure 7:**
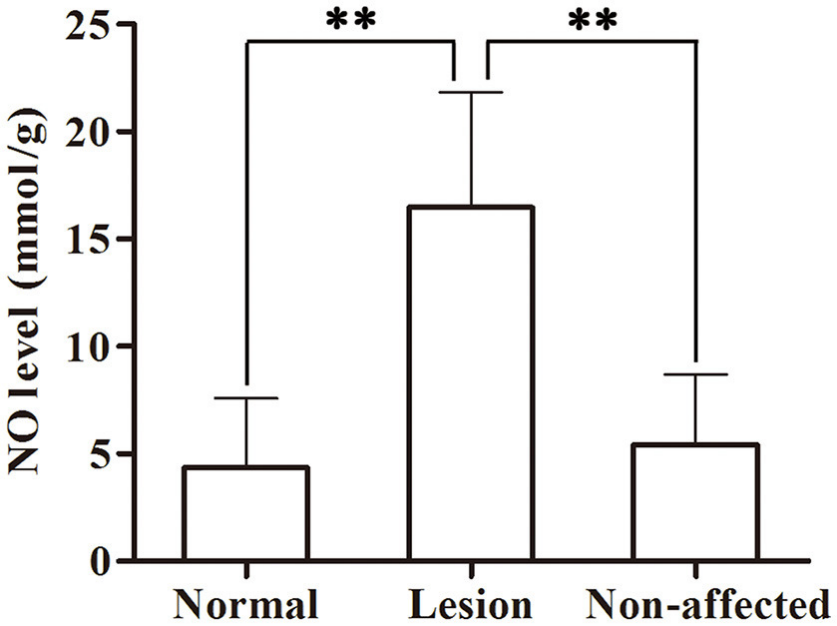
Skin tissue NO levels in a swine DCS model. Tissues were sampled in the lesion and non-affected skin from 11 swine after a simulated air dive to 40 m for 35 min with an 11 min decompression. Normal skin samples were collected pre-dive. NO was detected by ELISA. Values are presented as mean ± *SD* (^**^P < 0.01).

As shown in Figure 7, NO level increased significantly in lesion skin tissues compared to non-affected tissues (*P* = 0.002) or normal skins before modeling (*P* = 0.001). No difference was found between non-affected and normal skin (*P* = 0.611).

### Histology

Congestion was the most common finding in skin lesions, with red blood cells (RBCs) were observed clogging in the dermal capillaries. Other changes included dilatation, hemorrhage, and neutrophil infiltrates (Figures not present). In non-affected skin from the decompressed swine, only dilatation was occasionally observed. There was no abnormal findings in normal control skin. A summary of histologic findings is shown in Table 3.

**Table 3:** Summary of histologic findings.

**Anomaly**	**Skin lesion**	**Non-affected skin**	**Normal skin**
Congestion	11/11	0/11	0/11
Dilation	9/11	3/11	0/11
Neutrophil infiltrates	5/11	0/11	0/11
Hemorrhage	3/11	0/11	0/11

### CNS function

**Figure 8:**
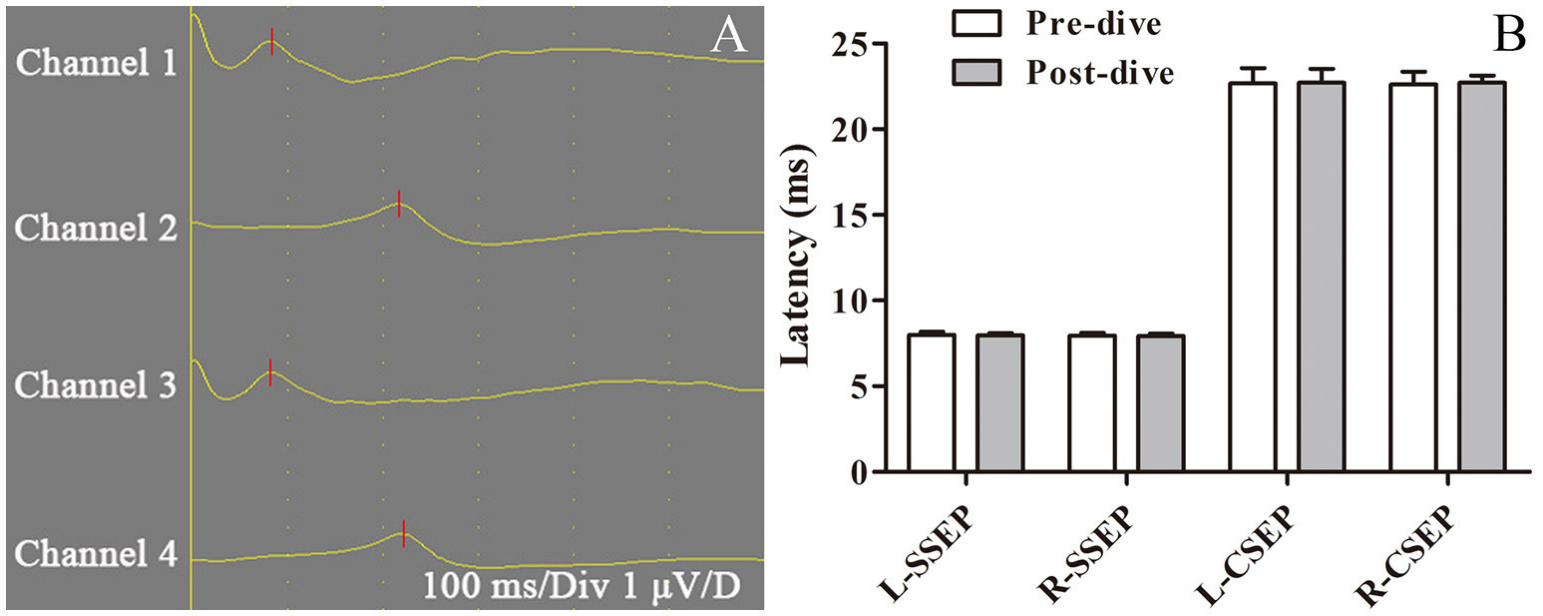
Sensory evoked potential in a swine DCS model. Sensory evoked potential (SEP) was tested pre-dive and 6 h post-dive in 11 swine following a simulated air dive to 40 m for 35 min with an 11 min decompression. First lumbar vertebra and head were selected to collect electroneurographic signals from right and left ankles, which were defined as spinal somatosensory evoked potential (SSEP, channels 1 and 3) and cortical somatosensory evoked potential (CSEP, channels 2 and 4) **(A)**. Both sides of SSEP and CSEP were compared pre-dive and post-dive, with no significant changes **(B)**.

Assessment of motor function at 2 and 6 h after decompression yielded a score of 5 in all survivors. Pre-dive and post-dive SSEP and CSEP were compared, with no significant changes (*P* > 0.05, Figure 8). Histological examination of spinal cord was also performed with no abnormal findings (data not shown). No other neurological abnormality was observed.

## Discussion

Swine have a number of anatomical and physiological similarities with humans that make them potentially a better model for procedures and studies than other large animal species (Smith and Swindle, 2006; Swindle et al., 2012). Therefore, swine have been employed in a wide range of diving related experiments (Powell et al., 1974; Dick et al., 1997; Bai et al., 2009). This study used 13 Bama swine reared for research purposes. The hair color is mostly white and the size of the animal is convenient relatively for handling in a laboratory. Their pale skin is an advantage for detecting cutaneous lesion changes.

DCS is caused by bubbles that are formed as a result of reduction in surrounding pressure (Vann et al., 2011). Resulting from the mechanical, embolic, and biochemical effects of bubbles, symptoms range from minor skin irritation to neurological symptoms, cardiac collapse, and even death (Neuman et al., 1980). Skin lesions are visual clinical manifestations, which to some extent reflect the condition of DCS. Although skin symptom cannot fully represent the severity of the whole body in clinical, it is still believed that early onset of cutis marmorata is sometimes followed by onset of neurological symptoms (Buttolph et al., 1998; Germonpre et al., 2015). All studies of swine DCS referred to skin lesions as Type I DCS and most simply describe lesions in general terms (Bai et al., 2009; Mahon et al., 2013, 2015).

The pathogenesis of cutaneous DCS remains controversial and three main perspectives exist: autochthonous bubbles, arterial bubbles embolizing the skin, and arterial bubbles embolizing the brain causing the skin symptoms via autonomic nervous system (Lambertsen, 1989; Wilmshurst et al., 2001; Hugon, 2014; Germonpre et al., 2015).

It has long been assumed that bubble formation in the skin tissue or skin capillaries may be the initiating mechanism but it is still in the absence of any substantial evidence (Buttolph et al., 1998; Hugon, 2014). Histology shows that the dermis is rich in lymphatic vessels, while subcutaneous tissue, closely connected with the dermis, is rich in adipose tissue, which is prone to form bubbles after decompression from hyperbaric exposure. In skin and subcutaneous tissues, supersaturated nitrogen diffuses into the venous system through capillaries in dissolved form or as micro bubbles. These bubbles pass into the venous system and ultimately to the right heart (Lambertsen, 1989). Although bubble detection has poor specificity or poor predictive value for clinical diagnosing of DCS, it is still the most objective way reflecting decompression stress, that is, inert gas load and risk of DCS following a dive. The present results indicate that skin lesions are associated closely with the appearance of venous bubbles. The faster occurrence, larger appearance, and longer existence of cutaneous lesions suggest a greater amount of bubbles formed inside the body.

The observed increase in NO in lesion tissue in this study may contribute to the vasodilatation in subcutaneous tissue, which was possibly the consequence of local bubbles diffused into capillaries, stimulating the endothelium (Palmer et al., 1987). Although tiny arterio-vein was extremely thin with slow blood flow, we still found local blood flow increasing with vasodilatation in subcutaneous tissue in the area around the lateral neck. These vessels, branch out into the capillaries in dermis (Sonksen and Craggs, 1999), could indirectly suggest that similar vasodilatation might occur in skin.

Bubbles will expand or coalesce in the skin capillaries and may obstruct blood flow before distributing into the venous system (Lambertsen, 1989; Hugon, 2014). When lesions progressed to middle-late stage III, the purple-red did not fade with pressure, which was postulated to be due to local congestion by bubbles in capillaries and venules locally. With bubbles discharging in vessels, hyperemia, and congestion were gradually restored, and hence the symptoms subsided, as indicated by stages IV to VI signs, which might also be due to the decreasing of supersaturation and bubble formation in local tissues. The bubbles' mechanical presence and any related tissue damage could incite inflammatory responses, and circulating bubbles could also induce reactions at the blood vessel wall (Levin et al., 1981). Endothelial damage by bubbles can cause capillary leakage and impaired endothelial function promotes leucocyte-endothelial adherence (Boussuges et al., 1996; Martin and Thom, 2002). Circulating bubbles interact with blood components and the endothelia triggering inflammation, multiple mechanisms and vasoactive mediators may play a significant role (Francis and Mitchell, 2003). All these effects cause an inflammatory exudation, thickening skin found in this study. Similar changes were found in a previous study focused extensively on the morphological changes (Buttolph et al., 1998).

Arterial bubbles embolizing in the subcutaneous capillary plexus was also proposed as the possible etiology of cutis marmorata (Wilmshurst et al., 2001). The presence of a large PFO and right-to-left shunt, even the bubble emboli passing through an overloaded lung filter, were considered a strong correlation with skin lesions (Wilmshurst et al., 1989, 2001; Cantais et al., 2003). The arterial bubbles will not only embolize in the subcutaneous capillary plexus, but also cause local disturbances of blood flow with subsequent inflammatory response (Wilmshurst et al., 2001; Conkin et al., 2002). However, in this study, although numerous bubbles were detected in the right heart, no bubbles were found in the left heart and all swine had complete structure of ventricular septal and interatrial septum, and no patent foramen ovale and no abnormal blood flow were observed.

One theory on the origin of skin DCS suggests that marbling is the result of skin blood vessel dilation and constriction abnormally regulated by the autonomic nervous system, which is driven by neurons located in the rostral ventromedial medulla of the brainstem close to the formatio reticularis (Germonpre et al., 2015). Cerebral air embolism has been found causing skin rash in swine by injecting air into cerebral circulation, but no rash has been found to occur with arterial bubbles in cerebral circulations in divers (Romero et al., 2009; Kemper et al., 2015; Wilmshurst, 2015). Although the brainstem was not investigated directly, no evidence in the current study indicated CNS dysfunction in motor function and sensory conduction, which could indicate brainstem dysfunction to a certain extent. Hence the current results do not support CNS dysfunction theory.

However, the lack of evidence in this DCS model still cannot rule out the arterial bubbles or CNS injury hypothesis. Small amount of bubbles could most possibly hide the detection, and CNS dysfunction indeed can cause cutaneous signs (Blogg et al., 2014; Kemper et al., 2015). Whether the CNS and arterial bubbles are involved in skin marmorata, especially in abrupt and large lesions in acute death, has yet to be studied.

The evolution of skin symptoms in previous swine studies remains to be fully described. In this study six stages of skin lesions, generalized from the appearance and the evolution of lesions, are described for the first time, which could help clearly identify the progress of the symptoms. Initially symptoms showed as stageI and II, some lasted up to 1 h, but some evolved to stage III within minutes. The lesions culminated in stage III, the most serious and largest symptom, and lasted for around 100 min. Then symptoms subsided slowly for several hours from Stage IV to VI until the lesions disappeared. Though the staging system is for swine DCS model, it may still serve to some extent as a reference for divers.

To limit the number of animals involved, the exposure profile was selected so that mortality was anticipated to be as low as possible and yet skin symptoms as severe as possible. Milder symptoms might not be induced by the current profile; hence, the grading of the manifestation may not fit all kinds of DCS skin lesions.

Although bubble scores were highest at the first detection and decreased gradually thereafter. The peak of bubbles most possibly appeared before the first detection. In future studies detection should start earlier and finish later. However, in this study earlier start of bubble detection would have stimulated the animal and increased the risk of mortality substantially (experience from our previous experiment) using the current chamber system. A new chamber under construction with a faster delivery system designed specifically for large animals will permit detection within 5 min following decompression. Bubble detection was not continued beyond 6 h due to the necessary oxygen breathing during anesthesia for SEP determination at 6 h following decompression, right after the last bubble detection. Motor evoked potential evaluation was not performed in this study to avoid invasive interventions to the skull or cortex, which may affect the other experiment procedures. Based on a series of inflammatory reactions caused by local bubble stimuli, we speculated that temperature might change in lesion skin, but we observed no positive confirmation of this hypothesis. Inflammatory reactions caused by local bubbles were slighter than common infection or trauma, for which fever is not usually significant.

In conclusion, this is the first study to systematically elucidate the clinical appearance and give strong evidence to support autochthonous bubbles as the etiology of skin lesions in a swine DCS model. Swine offer a good, or possibly even the best, animal model for the study of cutaneous DCS. Skin symptoms are easier to induce in swine than in human beings, together with the correlation with bubbles, may help assess the general decompression stress. Although the possibility cannot be ruled out, CNS dysfunction and arterial bubbles involvement was not observed.

## Author contributions

WX, LQ, and DA designed and LQ, DA, HY, YW, and QZ conducted the experiments. All authors listed contributed to data analyses and interpretation of the results. WX, LQ, and DA wrote the manuscript. WX, LQ, and DA prepared all the figures and the table. LQ and DA contributed equally to this work. All authors reviewed the manuscript and agreed to be accountable for the content of the work.

### Conflict of interest statement

The authors declare that the research was conducted in the absence of any commercial or financial relationships that could be construed as a potential conflict of interest.
